# Innovative Approaches to Fungal Food Production: Mycelial Pellet Morphology Insights

**DOI:** 10.3390/foods12183477

**Published:** 2023-09-19

**Authors:** Chih-Yu Cheng, Yu-Sheng Wang, Zhong-Liang Wang, Sidra Bibi

**Affiliations:** 1 Department of Marine Biotechnology, National Kaohsiung University of Science and Technology, Kaohsiung 81157, Taiwan; 0970yusheng@gmail.com (Y.-S.W.); tom3068162@gmail.com (Z.-L.W.); sidrajenifer@gmail.com (S.B.); 2 Institute of Aquatic Science and Technology, National Kaohsiung University of Science and Technology, Kaohsiung 81157, Taiwan

**Keywords:** Plackett-Burman design, Taguchi method, mycelial pellets, ImageJ, edible fungi, future food

## Abstract

Mycelia products enhance edible mushrooms in alignment with future sustainability trends. To meet forthcoming market demands, the morphology of mycelial pellets was optimized for direct consumption. Among ten commercial edible mushrooms in Taiwan, *Pleurotus* sp. was selected for its rapid growth and was identified via an internal transcribed spacer sequence. A combination of Plackett-Burman design and Taguchi’s L9(3^4^) orthogonal table revealed the optimal formula as potato dextrose broth (2.4%), olive oil (2%), calcium carbonate (0.5%), yeast extract (0.75%), and soy flour (0.5%). This led to a biomass increase to 19.9 ± 1.1 g/L, resulting in a 2.17-fold yield increase. To refine morphology, image processing by ImageJ quantified spherical characteristics. The addition of 0.2 to 1.0% Tween 80 enhanced pellet compaction by over 50%. Dilution of the medium improved uniformity (0.85) and conversion rate (42%), yielding mycelial pellets with 2.10 ± 0.52 mm diameters and a yield of 15.1 ± 0.6 g/L. These findings provide an alternative evaluation and application of edible mycelial pellets as future food.

## 1. Introduction

Owing to global environmental changes, indoor farming or speeding up the food production process is the development goal of modern food technology. The standard methods for cultivating edible mushrooms include solid-state and submerged liquid fermentation (SLF). The former produces the fruiting bodies of mushrooms, whereas the SLF produces mycelia. For the reason that culture parameters can be easily adjusted in SLF, production time is shorter and less wasteful than cultivation in traditional mushroom bags. It is also possible to stop tree-cutting and create mushroom bags. SLF makes the production of edible fungi more consistent with future trends suggested by sustainable development and environmental protection goals. As a result, edible fungal mycelia have emerged as a promising and sustainable food source for long-term human development. As well as meat analogs, products using fungal mycelia as health foods continue to receive attention and development [1,2]. This also means that increasing the production of mycelia and changing the color, flavor, taste, and shape of fungi will be a future trend in fungal foods.

Fungal hyphae display different morphologies, including filaments, clumps, and pellets, when cultured in SLF [3]. The mycelial pellet had a spherical shape formed by the winding of the mycelia. Due to the advantages of accessible collection and the lower viscosity of the fermented broth, which increases nutrient transfer and yield and reduces equipment loss [4], several applications of mycelial pellets have been developed in recent years. In addition to facilitating the recovery of fungi and their products [5], other microorganisms, such as algae, can be adsorbed and harvested by the flocculation of mycelial pellets [6,7]. They are also capable of adsorbing water pollutants, including heavy metals, cadmium (Cd) [8], pigments, and organic compounds [9].

However, the formation and morphology of fungal mycelial pellets are affected by numerous factors, including the fungal growth environment (temperature, stirring speed, and light) and medium conditions (carbon and nitrogen sources, minerals, surfactants, and oxygen). Fungal [10,11] and genetically modified species [12,13] can determine whether mycelial pellets are formed. Therefore, effectively determining appropriate culture conditions under such variables is challenging for each strain. To distinguish between these factors, numerous experiments must be conducted using classical experimental design methods. A well-planned experimental design is required to address this issue. 

Two experimental design methods are introduced here: the Taguchi method and the Plackett-Burman design (PBD) [14]. The PBD experimental design is typically used in the initial stage of the experiment, mainly to compare the difference between the two levels of each factor and the overall difference to judge the influence of the factor. This can avoid wasting resource costs caused by excessive control or insignificant factors in the subsequent optimization process. Taguchi methods are used to improve quality through statistical engineering optimization. An appropriate orthogonal array and signal-to-noise (S/N) ratio, which combines the mean and variance, were selected according to the product characteristics and control factors. After considering both productivity (mean) and stability (variance), an optimal combination was obtained.

Mushrooms, a popular type of edible fungus, can grow as mycelia in SLF. In the past, most SLF mushroom mycelia have been employed for subsequent culture inoculation [15] through dispersion of the mycelia or particles [16]. The main applications of mycelia in the food market have recently been focused on extracting their bioactive compounds and utilizing them in nutraceuticals and food flavors [17,18]. Although filamentous fungi are expected to be recognized as functional food and feed in the future [19] and their pellet formation has been well studied in industrial settings, there has been limited research directed towards mushroom mycelial pellets on their physical properties or for direct consumption in food markets. In our previous research, several vegan ingredients were identified for managing pellet quantity and size [20]. It would be beneficial to extend the vegetarianism concept and focus on enhancing the visual appeal of mycelial pellets to assess their potential as food for direct consumption. 

The current study combined two experimental design methods and image quantification to identify culture conditions that can quantify mycelium particles and change mycelium shape through several influencing factors. Precisely, a fast-growing edible mushroom was screened from ten commercial mushrooms in Taiwan. PBD was used to evaluate ten possible influencing biomass parameters. The Taguchi method was used to determine the optimal concentrations of the four media components and the optimal formula. Finally, the morphology of the pellets was adjusted using Tween 80 and medium strength and prepared as food products for the pioneer questionnaire to observe their potential. 

## 2. Materials and Methods 

### 2.1. Strain, Solid-State Culture, and Base Submerged Culture

Ten mushroom species were purchased from local markets, and their fruiting bodies were washed with sterile water and air-dried for 10 min. The stalks were cut into approximately 0.3 cm and inoculated onto potato dextrose agar (PDA) plates (potato dextrose broth [PDB], 24 g/L; agar, 20 g/L). After 5–10 days of growth at 25 °C, a 0.8 cm diameter mycelial piece was transferred to 100 mL of the basal medium (PDB with 0.5% yeast extract [YE]) and incubated on rotary shakers agitating at 130 rpm at 25 °C for 7 days. 

### 2.2. Strain Identification by Internal Transcribed Spacer (ITS) Sequencing 

A small quantity (1 mg) of mycelia was picked from the PDA plate and transferred into a 1.5 mL microcentrifuge tube containing 100 μL of pure water. Ten microliters of lysis solution (0.5 M NaOH) [21] were added, and the mixture was vortexed and incubated at 95 °C in a heating block for 10 min. During this time, the lysate was ground using a pestle (precisely fitted in the tube) driven by an electric drill driver at 1200 rpm for 1 min [22]. One µL of the supernatant was then used for PCR. 

PCR was carried out in 20 μL reactions containing 1 μL template, 1 μL of each 10 μM forward primer (ITS1F-CTTGGTCATTTAGAGGAAGTAA) and reverse primer (ITS4-TCCTCCGCTTATTGATATGC), 6 μL of 2× ToolsTaq PCR MasterMix KTT-BB01 (Biotools Co., Ltd., New Taipei City, Taiwan), and 10 μL of PCR-grade water to a total volume of 20 μL. After amplification, PCR products were checked on a 1% agarose gel and sequenced by a local biotechnology company (Mission Biotech Co., Ltd., Taipei City, Taiwan) (http://www.missionbio.com.tw (accessed on 17 September 2023). Sequences obtained from the forward and reverse primers were aligned. The aligned sequence was subjected to a homology search using NCBI BLAST to identify the genus and, if possible, species. 

### 2.3. Plackett-Burman Experimental Design

Ten factors, including nine additional nutrient sources, inoculation ratios, and one dummy factor, were selected for the PBD experiments. The test factors and their concentrations at different levels are listed in Table 1. According to the PBD N12 matrix, 12 experimental trials containing 100 mL of PDB medium and various concentrations of nutrient supplements were set up in 250-mL Erlenmeyer flasks. After sterilization by autoclaving at 121 °C for 15 min, one or two 0.8-cm-diameter pieces of mycelia were transferred into the flask. Then the flasks were incubated at 130 rpm agitation speed at 25 °C for 7 days. All the experiments were performed in triplicate, and the average values are reported as mean ± standard deviation (SD).

### 2.4. Taguchi Experimental Design 

Four additional nutrient sources—olive oil, calcium carbonate (CaCO_3_), yeast extract, and soybean flour—were added to the PDB medium, and their concentrations were adjusted using the Taguchi method. The three levels of these control factors in Table 2 and their compositions in the nine experiments were designed based on the Taguchi L9 orthogonal array. The flasks were inoculated with one 0.8-cm-diameter piece of mycelia and incubated at 130 rpm at 25 °C for 7 days. The S/N ratios for the nine trials were calculated for the larger-the-better quality characteristics as follows:

(1) SN=−10log⁡1n∑1yi2

where *y_i_* is the quality characteristic, and *n* is the number of repetitions performed for each experiment in the orthogonal array.

The contribution ratio for each factor, which provides the impression of its impact on production, is defined as the relational variation between the factorial effect and overall variation expressed as a percentage (that is, SSi/SST × 100%). 

### 2.5. Morphological Characterization

SLF broth was filtered through a sieve filter (16 mesh) to separate the mycelial pellets from the filtered medium. The mycelial pellets were washed with sterile water, analyzed by capturing images with a digital camera (Canon EOS M100; Canon Inc., Tokyo, Japan), and measured using ImageJ software v 1.48 [23] with parameters set to infinity particle sizes of 0.01 and roundness of 0.1–1.00. The morphologies of all the pellets in each sample were characterized by measuring the counts, area (A), perimeter (p), and Feret’s diameter. Circularity was estimated using 4πA/p^2^, and Christiansen’s uniformity coefficient value is presented as [24]:

(2) $$\mathrm{CU}=\left(1-\frac{\sum_{i=1}^{n}\left|xi-\left.\bar{x}\right|\right.}{n*\bar{x}}\right)$$

where xi is the Feret diameter of each pellet, $\bar{x}$ is the average Feret diameter, and n is the number of pellets.

### 2.6. ImageJ Software Analysis

ImageJ software v 1.48 was used to calculate the size, number, and morphological characterization of pellet images. Each sample photograph was uploaded into the ImageJ software v 1.48, and counts and Feret’s diameter of whole mycelial pellets were calculated. Christiansen’s uniformity coefficient value and circularity were analyzed using the above formula.

### 2.7. Dry Cell Weight (DCW) and Protein Assay

SLF broth was filtered through a sieve filter (16 mesh) to separate the mycelial pellets from the filtered broth. The mycelial pellets were analyzed for dry cell weight (DCW) by placing the pellets in an oven at 50 °C and recording weight daily until there was no change or 5–6 days. By adding an equal amount of sterile water to the mushroom samples, the mycelial pellets were homogenized at high speed for 3 min using a high-power blender (Vita-Mix Co., Olmsted Township, OH, USA), followed by analysis of the active ingredient content. Protein concentrations were determined using the Bradford method with BSA as the standard reference.

### 2.8. Polyphenol Determination 

The total phenolic content of the mycelia or broth was analyzed using a modified Folin-Ciocalteu method [25]. In brief, 60 µL of the sample was mixed with 300 µL of FC reagent (1/10×) and allowed to stand for 5 min; then, 240 µL of 16% Na_2_CO_3_ was added, mixed evenly, reacted at room temperature for 30 min in the dark, and measured at OD750 nm.

### 2.9. Total Polysaccharides Assay 

Total exopolysaccharides were precipitated by adding four volumes of 95% ethanol [26] and analyzed using the phenol-sulfuric acid method [27] for the whole carbohydrate assay. Next, 200 μL of 95% ethanol was added to 50 μL of each sample. After centrifugation at 12,000 rpm for 5 min, the precipitate was washed twice with 75% ethanol and dried at 37 °C for 15 min. The precipitate was dissolved by adding 200 µL of double-distiller water (ddH_2_O) and mixing thoroughly. The precipitate was then combined with 100 µL of 5% phenol solution and 500 µL of H_2_SO_4_ and incubated at 100 °C for 25 min. After cooling, the absorbance was measured at 490 nm.

### 2.10. Statistical Analysis

All experiments were performed in triplicate unless otherwise specified. Data are expressed as means ± SD and were calculated using MS EXCEL 2016 version. One-way analysis of variance (ANOVA) was used to test for significant factors after the PBD test. Statistical comparisons were made based on *p*-values (α = 0.05).

## 3. Results and Discussions

### 3.1. Fungal Strain and Growth Curve

**Figure 1:**
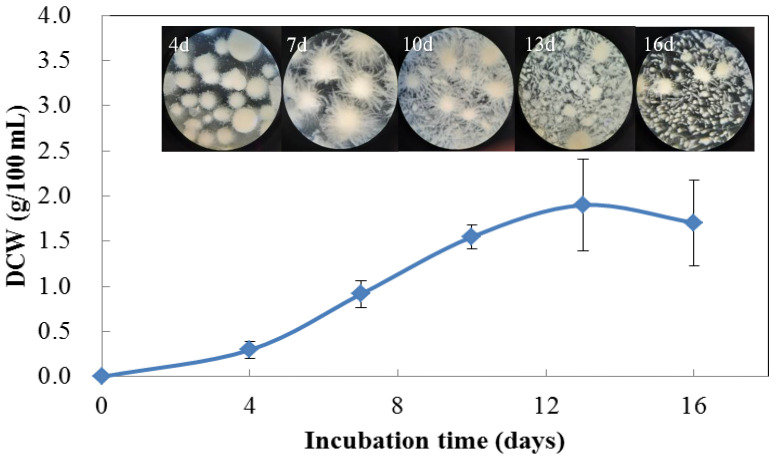
Time profile of biomass accumulation of mycelial pellets in submerged culture and morphological changes shown using a stereomicroscope.

To ensure the edibility of mushrooms, their suitability for public consumption, and the possibility of cultivation within the region, our research team selected ten strains of mushrooms from local supermarkets in Taiwan for evaluation. Their isolated mycelia were cultured on PDA plates for 5 days. Based on the size of the hyphal circle (Figure S1), the fastest-growing fungus was selected. Its two ITS sequences (NCBI accession numbers: OR512537 and OR512538) indicated that this dikaryotic mycelium contains two different haploid forms and was preliminarily identified as *Pleurotus* sp. (Figure S2). The mycelial growth results for different periods based on base-submerged culture conditions are shown in Figure 1. The DCW was in a straight line within 10 days and peaked at approximately 13 days. However, the appearance of the mycelial pellets changed from intact granular to burr-like, and they finally disintegrated into different sizes. The reasons for the fragmentation of mycelial particles are presumed to be low dissolved oxygen due to poor media rheology [28], the autolysis phenomenon [29], or friction due to a lack of space for high-cell-density cultivation. Therefore, 7 days was used under the following cultural conditions:

### 3.2. Factor Screening Using Plackett-Burman Design 

**Figure 2:**
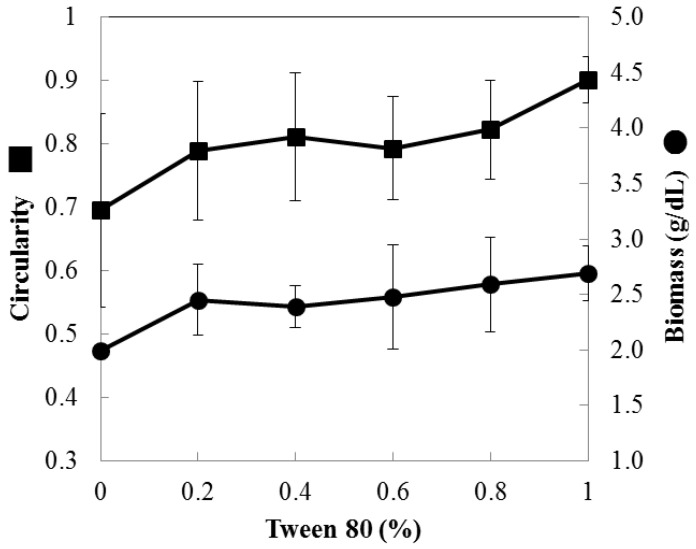
The effects of Tween 80 on circularity and biomass. Mycelial pellets were cultured in optimized formulation (A2B3C3D3) with different concentrations of Tween 80.

To optimize biomass production, 10 experimental factors were evaluated in 12 experimental PBD trials, and their average biomass values are shown in Table 3. These data were first divided into level (+) and level (−) groups, and the difference between the average values of the two groups was obtained as the effective value of each factor (Figure 2), calculated as

Effect = [∑ Y_(+)_/n_(+_)] − [∑ Y_(−)_/n_(−)_] (3)

A positive value indicates a positive influence at level (+1), that is, a high concentration of the component; otherwise, it has a negative influence. The extent of this effect (Figure S3B) was the highest with calcium carbonate (24%), followed by olive oil (20%), Tween 80 (19%), and KH_2_PO_4_ (13%), which also had the most considerable negative impact at higher concentrations (Figure S3A). 

A statistical ANOVA was used to compare the variations between the effects of each factor and background error to determine whether there was a significant effect. As shown in Table 3, the sum of the squares for the dummy factor is 0.012. Each factor (X1–X10) is compared with this estimated random error using a one-tailed F-test at the α = 0.05 significance level. The critical value of F_1, 1_ at α = 0.05 is 161; therefore, the effects of factors X4, X5, and X8 are significant. Collectively, PBD showed that calcium carbonate, olive oil, and Tween 80 had significant positive effects on the biomass production of mycelial pellets.

Three significant influencing factors screened by PBD exhibited similar results in previous studies: calcium carbonate [30], olive oil [31], and Tween 80 [32], all of which have been shown to support the production of fungal mycelia. However, other factors such as lignin [33], soybean flour [34], potassium dihydrogen phosphate [35], mung soybean flour [36], yeast extract [37], and inoculum ratio [38] are also promising factors for mycelial growth in SLF. This may be due to the diverse adaptability of different strains to the mixing action of various additives. Under a rationalized experimental design, although some valid factors may not be considered by PBD, it provides a quick method to compare the degree of influence of variables with limited time and resources. 

### 3.3. Taguchi Experiment Results

Four factors, including calcium carbonate and olive oil, yeast extract, and soybean powder, were selected for the Taguchi experiments to adjust the concentration of the optimal combination recipe. Calcium carbonate and olive oil are the top two most influential factors in PBD; yeast extract serves as an additional component of the basal medium to PBD medium; and soybean powder acts as an emulsifier substitution for Tween 80. Based on the three-level settings shown in Table 2, the experimental configuration was designed as shown in Table 4. The biomass results of mycelia production were affected by these four factors on biomass production, ranging from 0.65 to 2.11 g/dL. 

After grouping, the S/N ratio for each group was obtained (Table 4). This indicates that calcium carbonate contributed the most (~41%), followed by olive oil (30%), yeast extract (23%), and soybean flour (5%). The experimental results are consistent with the ranking results of the PBD. The S/N results at the factor level also indicated that the optimum combination was A2B3C3D3. These values represent the levels corresponding to those in Table 3. The optimal formula was 2% olive oil, 0.5% calcium carbonate, 0.75% yeast extract, and 0.5% soybean flour in PDB.

Under the optimal combination formula, the average biomass of mycelial pellets was increased from 0.92 ± 0.15 g /dL (basal medium) to 1.99 ± 0.11 g/dL (S/N = 6.0). The experimental S/N value is also consistent with the values predicted by the additive model (S/N = 7.5 ± 4.4, details in Tables S1 and S2). This confirms the validity of the Taguchi experiment in this study.

By combining the PBD and Taguchi experiments, this study successfully determined the optimal composition and ratios for 10 potential influencing factors. This resulted in a remarkable 2.2-fold increase in biomass production using only 21 formulated media. This innovative approach provided a practical method for identifying optimal formulations in future production processes.

### 3.4. Optimization of Mycelial Pellet Morphology Using Tween 80

During biomass optimization, these factors affect not only the biomass but also the shape of the mycelial pellets. Pellets with different morphologies were observed in various media using PBD and Taguchi methods. Although the biomass yield doubled, the optimized biomass formulation (A2B3C3D3) failed to support a better mycelial pellet shape (Figure S4A). To develop edible forms, the shape of the mushroom pellets must be optimized to increase their exploitation value. Therefore, other supplements or strategies are required to optimize the mycelial pellet morphology. 

The addition of Tween 80 tended to agglomerate filamentous mycelial debris (Figure S4). Notably, as the concentration of Tween 80 increased, circularity and biomass also exhibited an upward trend, reaching 29% and 35%, respectively, as illustrated in Figure 2. However, no significant impact was observed on the number and size of the particles (as detailed in Table S3). That is, in addition to improving sphericity, Tween 80 increased particle density and enhanced pellet compactness. Nevertheless, the persistence of non-uniform particle size (77–80%) in high-cell density cultures remains a challenge. 

The beneficial influence of Tween 80 on fungal growth is consistent with the findings of [32], who reported a favorable effect of Tween 80 on *P. eryngii* growth. They also discovered that Tween 80 resulted in the creation of mycelial micropores by inducing surface roughening. Whether this result is due to the amplified adsorption surface or to assist in olive oil emulsification, incorporating the surfactant Tween 80 contributed significantly to biomass enhancement. It is worth noting that the addition of Tween 80 did not lead to increased biomass for *Ganoderma* pellets in our laboratory. In fact, it also disrupted pellet formation and resulted in smaller and less compact *Cordyceps* pellets [39]. These conflicting outcomes suggest that further experimentation is required to understand the mechanisms by which Tween 80 affects pellet formation.

### 3.5. Optimization of Mycelial Pellet Morphology through Medium Intensity

**Figure 3:**
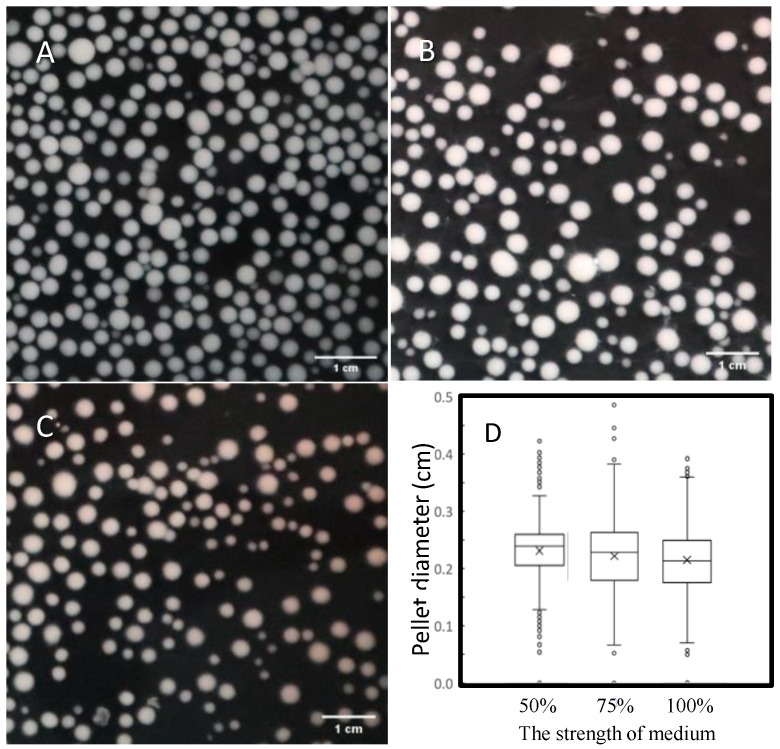
The effect of medium strength on pellet diameter. To accurately calculate the particle size and avoid particle overlap, the pellets cultivated in different medium strengths ((**A**): 50%, (**B**): 75%, and (**C**): 100%) were diluted appropriately, photographed, 444–519 particles were analyzed by the ImageJ and calculated as a box plot (**D**). The five-number summary is the minimum, first quartile, median, third quartile, and maximum. X indicates the average value and circles (○) for outlier data, determined by points lying further than 1.5-fold the interquartile range from the edge of the box.

The strength of the culture medium was reduced to 50% and 75% to avoid debris and mycelial pellets of different sizes due to high cell density, respectively. The conversion rate (biomass/material) increased from 2.69 ± 0.25 to 2.14 ± 0.12 and 1.51 ± 0.06 (Table S3), and the efficiency of the conversion rate (biomass/material) increased from 32% to 42%. In addition, less debris was observed in the low-intensity medium (Figure 3A) than in the full-intensity medium fermentation (Figure 3C), where numerous fragments leading to small pellets were observed. As shown in Figure 3D, the 50% intensity group was centered on a larger pellet and was more tightly distributed than the other groups. It was also determined that after autoclaving at 60 °C for 30 min, the mycelial pellets prepared in a 50%-strength medium maintained more pellet integrity than those produced in a higher-strength medium. The 50%-strength medium effectively reduced fragmentation, resulting in uniform pellet diameters, increased uniformity to 85%, and improved structural rigidity.

### 3.6. Assessment of Optimization and Food Performance 

Yield enhancement through PBD and the Taguchi method resulted in a 217% increase in biomass, which reached 294% with the additional 1% of Tween 80 (Table S3). The best mycelial pellet morphology, uniformity (85%), and conversion rate (42%) were obtained by reducing the medium intensity to 50%. Finally, 1.5 ± 0.06 g/dL of mycelial pellets with particle sizes of 2.10 ± 0.52 mm were achieved. Analysis of the active substances in different forms of *Pleurotus* mushrooms showed that the crude protein and soluble polysaccharide contents were similar to those in the fruiting body (Figure S5). In contrast, the total polyphenol content was lower than that of the fruiting bodies. In the realm of fungal mycelia, controlling morphology plays a key role in altering metabolic pathways for specific enzyme or product production. For example, previous studies have shown that mycelial pellet formation can enhance the production of mycophenolic acid [40] and fructofuranosidase [41]. Conversely, inhibition of pellet formation has been shown to be beneficial for EPS production [39]. This suggests that mycelia not only have the capacity to produce higher amounts of polysaccharides than fruiting bodies but also that, through appropriate adjustments in pellet morphology, the production of polysaccharides can be further optimized for efficiency.

Compared with traditional plant-based ingredients, fungal alternatives to animal-based meat offer various benefits, including a well-balanced essential amino acid profile, reduced waste generation, minimal competition for food resources, and a diminished environmental impact [42]. Despite ongoing developments in legislation and regulatory requirements, products utilizing *Fusarium* mycelium as a meat analog have quietly entered the market [43,44]. Additionally, in 2021, textured beverage products based on fungi such as *A. awamori* and *A. oryzae* were developed [1]. These mushrooms are often used to make fermented foods. It is worth noting that mushrooms, a type of fungus, have traditionally been consumed as food, particularly their fruiting bodies. This unusual quality could make it easier to get regulatory approval and ensure the safety compliance required for the development of edible mycelial pellets for direct consumption, making it an attractive and promising avenue for further research. 

A small questionnaire was carried out to understand the potential and market for direct consumption of this mycelium product. Mycelium pellets were first produced using the optimal culture conditions described above for 7 days. After filtering to remove the supernatant, autoclaving at 60 °C for 30 min, and simple seasoning with salt and sugar, the mycelial pellets were developed into savory and sweet products. The results of the pioneering questionnaire for local teachers and students showed an overall acceptance rate of 5.79 (10-point Likert scale). For the reason that the taste preferences of the participants varied widely (savory products at 45% and sweet products at 17%), optimizing the seasoning taste of pellet products is required in future studies. However, the complex relationship between mycelial metabolites and various aspects of the food, including safety, nutrition, color, aroma, taste, and flavor, warrants further investigation.

## 4. Conclusions

In this study, a combination of Plackett-Burman screening and Taguchi optimization experiments resulted in a twofold increase in the yield of liquid culture mycelium pellets. The optimization of mycelial morphology was achieved through image processing, which allowed for the quantification of spherical characteristics. By introducing Tween 80 and adjusting medium strength, improvements were made in the roundness of the spheres, uniformity of pellets, and biomass conversion rate, aligning with market demands for high-quality fungal products. However, further investigation is required into more detailed morphological parameters and the relationship between mycelial morphology and metabolites. 

## Figures and Tables

**Table 1:** Assigned concentrations of ten independent variables at different levels in PBD in submerged culture.

Symbols	Factors	Level 1 (+1)	Level 2 (−1)	Units
X1	Lignin ^1^	1	0	mL
X2	Inoculation	2	1	pieces
X3	YE	0.5	0.25	g
X4	CaCO_3_	0.5	0	g
X5	Olive oil	2	0	mL
X6	Yolk powder	0.5	0.25	g
X7	Soy powder	0.5	0.25	g
X8	Tween 80	1	0	mL
X9	KH_2_PO_4_	2	1	g
X10	Mung bean powder	0.5	0	g
X11	Dummy factors			

Note ^1^: 4 wt% waste lignin from local pulp mills.

**Table 2:** Control factors and selected levels in 100 mL of culture medium.

Symbols	Factors	Level 1	Level 2	Level 3	Units
A	Olive oil	0	2	4	mL
B	CaCO_3_	0	0.25	0.5	g
C	YE	0.25	0.50	0.75	g
D	Soy powder	0.00	0.25	0.50	g

**Table 3:** Medium formulations of PBD12 experimental trials along with DCW results and ANOVA.

Trial No.	X1	X2	X3	X4	X5	X6	X7	X8	X9	X10	X11	DCW
1	1	1	0.5	0	0	0.25	0.5	1	2	0	-	0.50 ± 0.23
2	1	2	0.25	0.5	0	0.25	0.25	1	2	0.5	-	2.35 ± 0.07
3	0	2	0.5	0	2	0.25	0.25	0	2	0.5	-	1.64 ± 0.91
4	1	1	0.5	0.5	0	0.5	0.25	0	1	0.5	-	2.25 ± 0.19
5	1	2	0.25	0.5	2	0.25	0.5	0	1	0	-	2.48 ± 0.31
6	1	2	0.5	0	2	0.5	0.25	1	1	0	-	2.81 ± 0.18
7	0	2	0.5	0.5	0	0.5	0.5	0	2	0	-	1.19 ± 1.01
8	0	1	0.5	0.5	2	0.25	0.5	1	1	0.5	-	4.12 ± 0.30
9	0	1	0.25	0.5	2	0.5	0.25	1	2	0	-	3.73 ± 1.28
10	1	1	0.25	0	2	0.5	0.5	0	2	0.5	-	0.58 ± 0.30
11	0	2	0.25	0	0	0.5	0.5	1	1	0.5	-	1.77 ± 0.38
12	0	1	0.25	0	0	0.25	0.25	0	1	0	-	0.94 ± 0.19
Effect ^1^	−0.401	0.021	0.111	1.313	1.06	0.049	−0.514	1.031	−0.729	0.176	0.062	
SS ^2^	0.483	0.001	0.037	5.173	3.37	0.007	0.791	3.19	1.593	0.093	0.012	
MS ^3, 4^	0.483	0.001	0.037	5.173	3.37	0.007	0.791	3.19	1.593	0.093	0.012	
F-value ^5^	41.95	0.11	3.2	449.6	292.9	0.63	68.78	277.3	138.5	8.11	1	
P ^6^	0.098	0.793	0.325	* 0.030	* 0.037	0.573	0.076	* 0.038	0.054	0.215	0.5	

Note: ^1^. Effect = [∑ Y_(+)_/n_(+)_] − [∑ Y_(−)_/n_(−)_], n(+) = n(−) = n/2 = 6. ^2^. SS: sum of square = n [(effect)^2^]/4, n = 12. ^3^. df = 1. ^4^. MS: mean of square = sum of square/degree of freedom = SS/df. ^5^. F-value = MSi/MS (dummy), F (1,1) = 161. ^6^. *: *p* < 0.05.

**Table 4:** Orthogonal array L9 matrix of Taguchi method and experimental results (n = 3).

	Factors	Olive oil	CaCO_3_	YE	Soy Powder	Biomass	S/N Ratio
Trial no.	L1	0.0	0.0	0.3	0.0	0.65 ± 0.16	−4.52
	L2	0.0	0.3	0.5	0.3	1.36 ± 0.09	2.61
	L3	0.0	0.5	0.8	0.5	1.71 ± 0.22	4.47
	L4	2.0	0.0	0.5	0.5	1.61 ± 0.28	3.75
	L5	2.0	0.3	0.8	0.0	2.11 ± 0.37	6.03
	L6	2.0	0.5	0.3	0.3	1.66 ± 0.14	4.29
	L7	4.0	0.0	0.8	0.3	1.27 ± 0.21	1.68
	L8	4.0	0.3	0.3	0.5	1.52 ± 0.31	3.03
	L9	4.0	0.5	0.5	0.0	1.81 ± 0.29	4.78
S/N ratio	Level 1	0.85	0.30	0.93	2.10		
	Level 2	4.69	3.89	3.71	2.86		
	Level 3	3.16	4.51	4.06	3.75		
	SS ^1^	22.38	30.97	17.63	4.09		
	Contribution ^2^	30%	41%	23%	5%		

Note: ^1^. SS: sum of square. ^2^. Contribution calculated using SSi/SST × 100%, SST = 75.06.

## Data Availability

The data used to support the findings of this study can be made available by the corresponding author upon request.

## Supplementary Materials

The following supporting information can be downloaded at: https://www.mdpi.com/article/10.3390/foods12183477/s1, Figure S1: The fruiting body and growing mycelium on PDA plate; Figure S2: The phylogenetic tree of ITS based on NCBI and ITS2 database; Figure S3: The factor effects and effects proportion of 10 independent variables of Plackett-Burman Design; Figure S4: Mycelial pellets morphology cultured in optimized formulation with different concentrations of Tween 80; Figure S5: Comparative analysis of biologically active substances in filtrate, mycelial pellets and fruiting body; Table S1: Response table and analysis of variance (ANOVA) of S/N ratio for biomass; Table S2: Optimal condition and prediction results of Taguchi; Table S3: Assessment and summary of optimization procedure.
