# Supplementary material for: Innovative Approaches to Fungal Food Production: Mycelial Pellet Morphology Insights
Source: Foods. 2023 Sep 19;12(18):3477. doi: 10.3390/foods12183477 (PMC10530132; doi:10.3390/foods12183477)
Supplement: Supplementary file 1 [file foods-12-03477-s001.zip › foods-2599452-supplementary.pdf]

Supplementary data

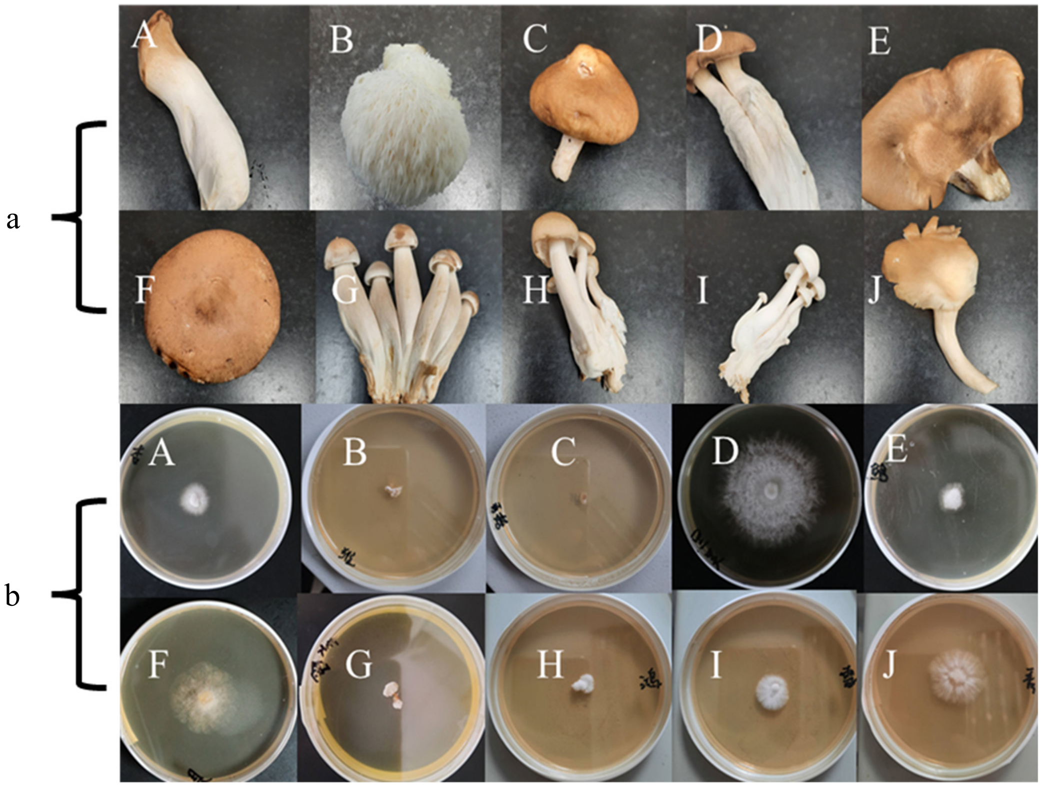

Figure S1 The fruiting body (a) and growing mycelium on PDA plate (b) of ten edible mushrooms including King trumpet mushroom (A), Lion's mane mushroom (B), Shiitake mushroom (C), Oyster mushroom-1 (D), Abalone mushroom (E), Table mushroom (F), Zhengjigu mushroom (G), Beech mushroom-1 (H), Beech mushroom-2 (I), Oyster mushroom-2 (J). The common name is based on the product description and mushroom appearance. Mushroom (D), observing the largest mycelium indicating the fastest growth, identified as *Pleurotus* sp. by ITS sequence and selected for in this study.

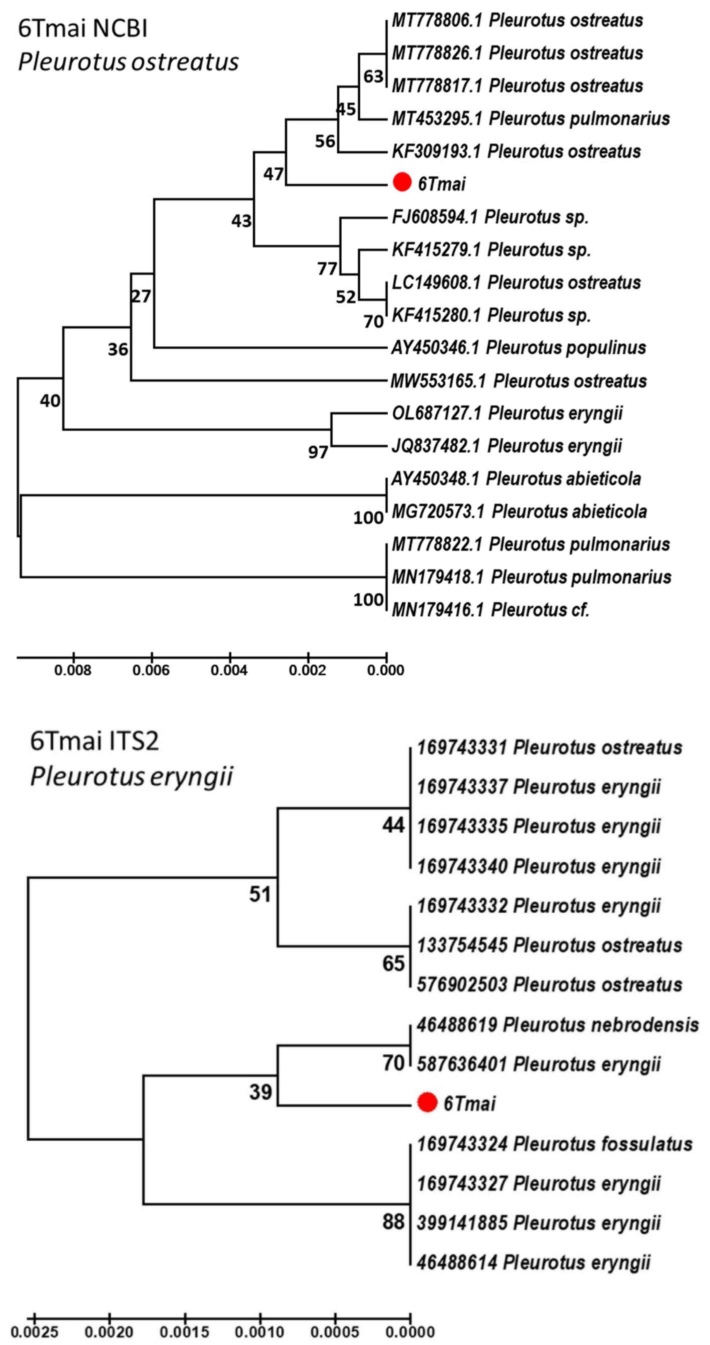

Figure S2 The phylogenetic tree of ITS 6Tmai based on NCBI and ITS2 database. This dikaryotic mycelium has two ITS isoforms (5Tmai and 6Tmai) and no reference species with more than 98.06% similarity on both databases. Besides, both sequences of 5Tmai and 6Tmai are BLAST as *Pleurotus ostreatus* in NCBI (https://www.ncbi.nlm.nih.gov/, accessed on 10 September 2023), but *Pleurotus eryngii* in ITS2 (http://its2.bioapps.biozentrum.uni-wuerzburg.de/, accessed on 10 September 2023). It has been suggested that ITS does not provide sufficient resolution between closely related species, and therefore secondary barcoding is required for species identification, such as translation elongation factor 1α (TEF1). Therefore, before conducting further experiments, we have decided to name the mushroom in this study *Pleurotus sp*. to avoid confusion.

(A)

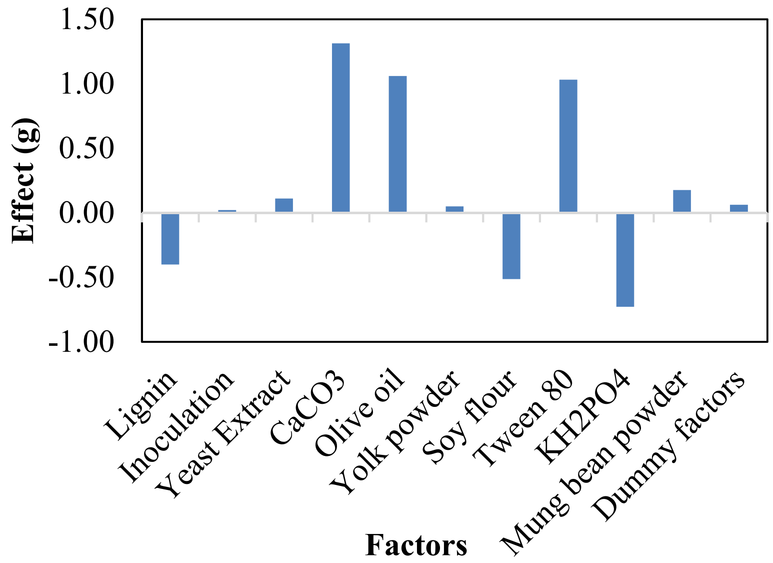

(B)

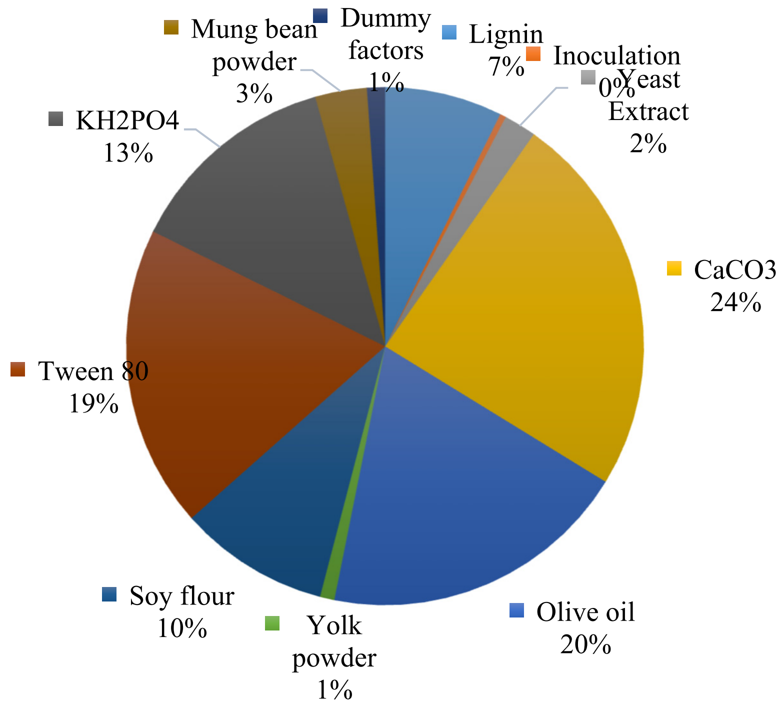

Figure S3 The factor effects (A) and effects proportion (B) of 10 independent variables of Plackett-Burman Design. A positive value (A) indicates a positive impact of level; otherwise, it is a negative influence.

Table S1 Response table and analysis of variance (ANOVA) of S/N ratio for biomass

**Factors** [*1]	**df**	**SS**	**MS**	**Contribution**[*2]	***F*-test**	**Contribution**[*3]
Olive oil	2	22.38	11.19	30%	5.47	24%
$CaCO_3$	2	30.97	15.48	41%	7.57	36%
YE	2	17.63	8.81	23%	4.31	18%
Soy powder	2	4.09	2.05	5%		
Error	2	4.09	2.05		1.00	22%
Total	8	75.06	9.38	100%		100%

Note:

*1: The variance of the factor with an open circle, with the most minor contributing, is pooled into the error factor.

*2: Contribution calculated by SSi/SST*100%

*3: Contribution calculated by (Yokoyama, 2003):

$$\rho(A) = \frac{SS_A - DFA * MS_{(e)}}{SS_T} *100\%$$

$$\rho(e) = \frac{SS_E + (DF_A + DF_B + DF_C + DF_D) * MS_{(e)}}{SS_T} *100\%$$

Yokoyama, S. (2003). Taguchi-style quality engineering lectures: Experimental planning method for quality design (2nd ed.). *China Productivity Center*.

Table S2 Optimal condition and prediction results of Taguchi

Factors	Olive oil	$CaCO_3$	Yeast extract	Soy powder
Opt. Level	2	3	3	3
Amount (g)	2	0.5	0.75	0.5
Max. S/N	4.7	4.5	4.1	3.7
Significant factor or not	yes	yes	yes	no
Prediction*1		S/N=8.301	; Y=2.6 g	
Prediction*2		S/N=7.455	; Y=2.359 g	; CI*3=4.402

Note:

*1: prediction by the additive model with all 4 factors.

*2: prediction by the additive model with 3 significant factors.

*3: A confidence interval was obtained by substituting MSe (2.05 in Table S1) to the following formula:

$$CI = \sqrt{F_{0.05;1,\nu} * MS_e * \left[\frac{1}{n_{eff}} + \frac{1}{r}\right]}$$

Where ν and MS(e) is freedom and mean square of error factor, n_{eff} = Total number of experiments/ (total number of freedom degrees of significant factor and +1), and r is the number of repetitions to confirm the experiment, r is 3 in this experiment.

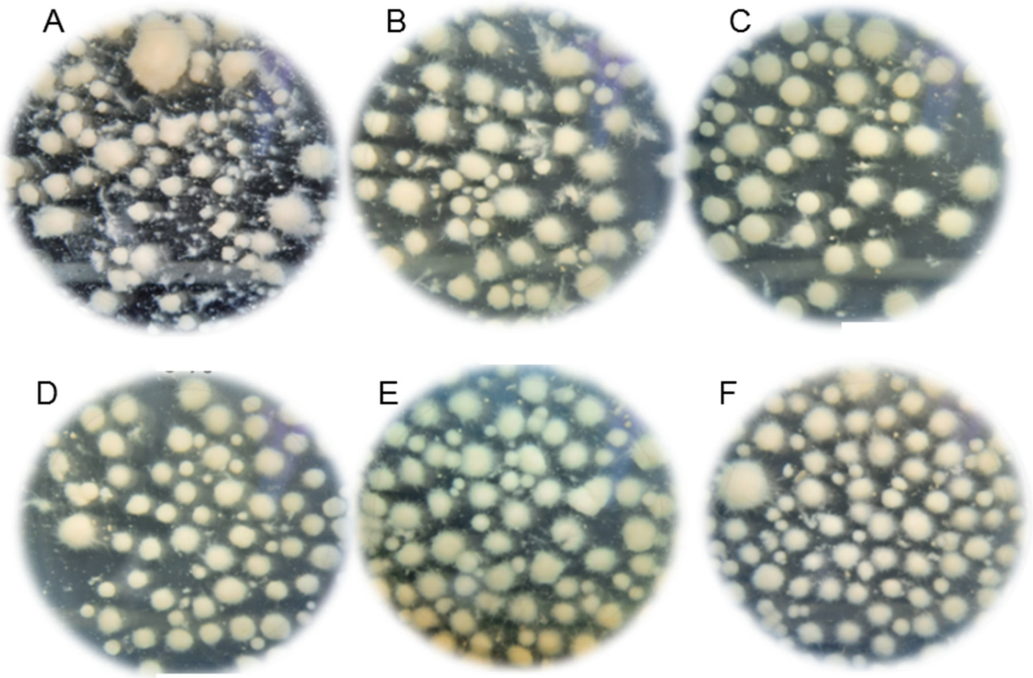

Figure S4 Mycelial pellets morphology cultured in optimized formulation (A2B3C3D3) with different concentrations of Tween 80. A~F represent Tween 80 concentrations of 0, 0.2, 0.4, 0.6, 0.8 and 1%. This suggests that more compact mycelium particles can be obtained using Tween 80.

Table S3 Assessment and summary of optimization procedure

Trial[*1]	Medium content						Production				Morphology		
	Olive oil	$CaCO_3$	YE	Soy flour	PDA	Tween 80	Biomass	Yield Enhancement[*2]	Conversion rate[*3]	Number of pellets	Diameter	Uniformity	Density[*4]
	mL	g	g	G	g	mL	g	%	%		mm	%	$10^{-6}g/mm^3$
**1**			0.5		2.4		0.92±0.15	100%	32%	128±32	8.5±1.9	84%	22
**2**	2	0.5	0.75	0.5	2.4	0	1.99±0.06	217%	32%	4104±1533	2.21±0.74	76%	86
**3**	2	0.5	0.75	0.5	2.4	0.2	2.45±0.32	268%	39%	4041±2247	2.39±0.70	77%	85
**4**	2	0.5	0.75	0.5	2.4	0.4	2.39±0.19	261%	37%	3509±1705	2.38±0.63	80%	96
**5**	2	0.5	0.75	0.5	2.4	0.6	2.48±0.47	271%	37%	4090±1477	2.18±0.59	78%	112
**6**	2	0.5	0.75	0.5	2.4	0.8	2.59±0.43	283%	37%	3940±2267	2.38±0.71	78%	93
**7**	2	0.5	0.75	0.5	2.4	1	2.69±0.25	294%	38%	5477±3257	2.12±0.64	78%	98
**8**	1.5	0.375	0.56	0.375	1.8	0.75	2.14±0.12	234%	40%	4310±414	2.30±0.60	82%	78
**9**	1	0.25	0.375	0.25	1.2	0.5	1.51±0.06	165%	42%	3415±454	2.10±0.52	85%	91

Note:

1. 1: basal medium; 2: Taguchi's optimal medium (A2B3C3D3); 2~7: experiments with different concentrations of Tween 80; 7~9: experiments with different medium strengths.
2. Yield enhancement = biomass/biomass of trial 1*100%
3. Conversion rate = material/biomass*100%
4. Density = biomass) *10^6/pellets number/($4/3\pi r^3$)

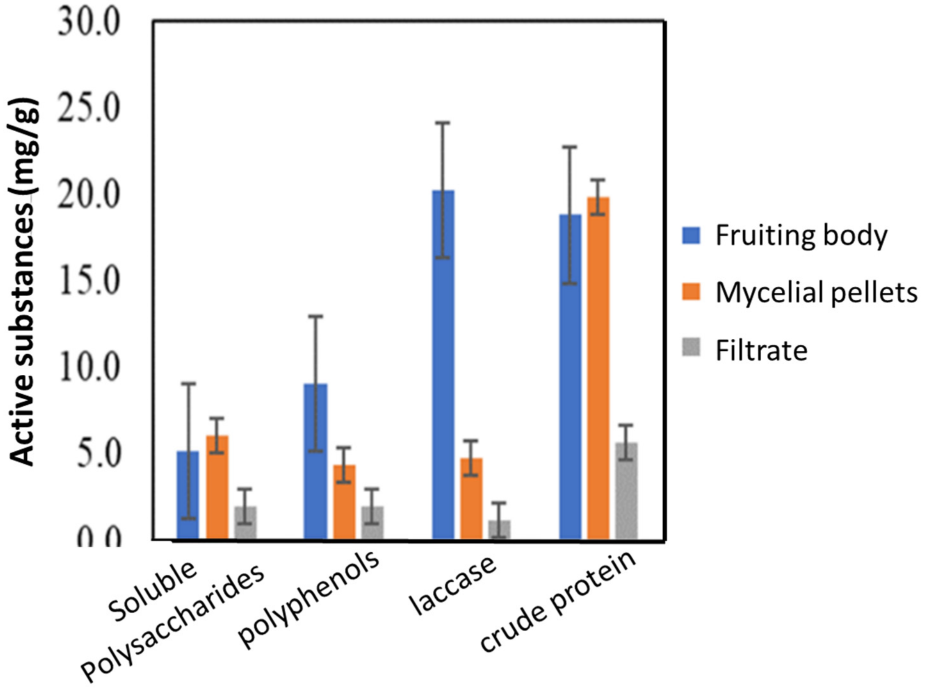

Figure S5 Comparative analysis of biologically active substances in filtrate, mycelium pellets, and fruiting body. The optimized mycelial pellets proved the stimulating effects as a result of polysaccharides level, crude protein and laccase activity.
